# A systematic review of the interventions to promote the wearing of hearing protection

**DOI:** 10.1590/S1516-31802007000600013

**Published:** 2007-11-01

**Authors:** Regina Paolucci El Dib, Álvaro Nagib Atallah, Régis Bruni Andriolo, Bernardo Garcia de Oliveira Soares, Jos Verbeek

**Affiliations:** Discipline of Emergency Medicine, Brazilian Cochrane Center, Universidade Federal de São Paulo (Unifesp), São Paulo, Brazil

**Keywords:** Ear protective devices, Noise-induced hearing loss, Occupational noise, Review literature, Meta-analysis, Dispositivos de proteção das orelhas, Perda auditiva provocada por ruído, Ruído ocupacional, Literatura de revisão, Metanálise

## Abstract

**CONTEXT AND OBJECTIVE:**

Noise-induced hearing loss can only be prevented by eliminating or lowering noise exposure levels. When the source of the noise cannot be eliminated, workers have to rely on hearing protection equipment. The aim here was to summarize the evidence for the effectiveness of interventions to enhance the wearing of hearing protection among workers exposed to noise in the workplace.

**DATA SOURCE:**

Studies with random assignment were identified by an electronic search of the medical literature up to 2005. Data were double-entered into the Review Manager software, version 4.2.5.

**DATA SYNTHESIS:**

Two studies were found. A computer-based intervention tailored to individual workers’ risks and lasting 30 minutes was not found to be more effective than a video providing general information for workers. A second randomized controlled trial evaluated the effect of a four-year school-based hearing loss prevention program among schoolchildren working on their parents’ farms. The intervention group was twice as likely to wear some kind of hearing protection as was the control group (which received only minimal intervention).

**REVIEWERS’ CONCLUSIONS:**

The limited evidence does not show whether tailored interventions are more or less effective than general interventions among workers, 80% of whom already use hearing protection. Long-lasting school-based interventions may increase the use of hearing protection substantially. Better interventions to enhance the use of hearing protection need to be developed and evaluated in order to increase the prevention of noise-induced hearing loss among workers.

## RESUMEN

**CONTEXTO E OBJETIVO:**

Perda auditiva induzida por ruído pode apenas ser prevenida eliminando ou diminuindo os níveis de exposição sonora. Quando a fonte do ruído não pode ser eliminada, trabalhadores devem utilizar os equipamentos de proteção auditiva. O objetivo foi sumarizar as evidências sobre efetividade das intervenções para aumentar o uso de protetores auriculares entre trabalhadores expostos a ruídos.

**ESTRATÉGIA DE BUSCA:**

Estudos randomizados foram identificados por busca eletrônica na literatura médica até 2005. Os dados foram checados duplamente e inseridos no software Review Manager software, versão 4.2.5.

**RESULTADO PRINCIPAL:**

Dois estudos foram encontrados. Uma intervenção padronizada por computador em relação ao risco de um trabalhador individual durou 30 minutos e não foi mais efetiva do que um vídeo fornecendo informações gerais entre os trabalhadores. Um segundo ensaio clínico randomizado avaliou os efeitos de um programa de prevenção auditiva de quatro anos em uma escola onde jovens trabalhavam nas fazendas de seus pais. O grupo de intervenção foi duas vezes mais provável de usar algum tipo de protetor auricular do que o grupo controle, que recebeu mínima intervenção.

**CONCLUSÃO DOS REVISORES:**

Limitada evidência não demonstrou quando as intervenções padronizadas são mais ou menos efetivas do que intervenções gerais em trabalhadores, 80% deles já utilizavam o protetor auricular. Intervenções longas em escolas podem aumentar substancialmente o uso de protetores auriculares. Melhores intervenções para aumentar o uso de Equipamentos de Proteção Individual (EPIs) devem ser desenvolvidas e avaliadas com intuito de aumentar a prevenção de perda auditiva induzida por ruído em trabalhadores.

## INTRODUCTION

Noise-induced hearing loss is one of the most common occupational diseases and the second most common self-reported occupational illness or injury. The condition is permanent and irreversible. Furthermore, there is no effective treatment for permanent hearing loss resulting from excessive noise exposure. However, the condition can be prevented by preventive measures, and sets of such measures are usually combined into hearing loss prevention programs (also called hearing conservation programs). Personal protective measures (e.g. earplugs or earmuffs) always form one part of a hearing loss prevention program.^1^ Even though the efficacy of these measures for shielding the inner ear from noise has been proven in laboratory settings, their effectiveness for preventing hearing loss from accumulated noise exposure depends mainly on how regularly they are used by workers. Studies have shown that if workers do not wear hearing protection for 100% of the time, its effectiveness will quickly diminish. For example, wearing hearing protection for only 90% of the time will decrease effectiveness to less than one third.^2^ Educational or behavioral interventions to promote its use are therefore important preventive measures.

### Prevalence and etiology

The risk of hearing loss due to noise exposure has long been recognized. Approximately 30 million workers in the United States alone are exposed to hazardous noise at work.^3^ Early damage is typically sustained in the basal turn of the cochlea and affects hearing in the frequency range from 3000 to 6000 Hertz (Hz) (the frequency range of speech). One study calculated an annual worldwide incidence of noise-induced hearing loss of 1,628,000 cases.^4^ With a worldwide population of 6.525 billion this is equal to 25 per 100,000 per year. Long-term exposure to noise levels greater than 80 dB(A) (i.e. situations in which you have to raise your voice if you want to communicate with someone who is within a distance of one meter) carries an increased risk of hearing loss, which increases exponentially with the noise level. The risk of hearing impairment (average hearing loss > 35 dB(A) at 1, 2 and 3 kHz) at age 60 due to 40 years of exposure to noise levels of 100 dB(A) has been estimated as 55%.^5^ Concurrent exposure to ototoxic substances, such as solvents and heavy metals, may increase the potential for damage from noise.^6^ Individuals’ susceptibility to the adverse effects of noise exposure is highly variable and cannot be accurately predicted.

### Prevention

Hearing loss prevention programs in industry have been widely advocated. Occupational health and safety legislation obliges employers to take preventive measures in most countries.^1^ These have proven to be effective to some extent in countries like Finland, where the incidence of cases of noise-induced hearing loss halved between 1987 and 2002.^7^ In the European Union and the United States, assessment of exposure to noise is obligatory, as is periodic screening of workers exposed to certain noise levels. Employers are also obliged to follow a "hierarchy of hazard controls". This is designed to eliminate hazards in the workplace in a particular order, by establishing controls at the source of the hazard (engineering or administrative) before using less reliable human controls (in this case the wearing of personal protective equipment). However, technical or economic reasons may mean that human controls are heavily relied upon. Indeed, a recent study has shown that personal protective equipment is still a widely used hazard control.^8^ From laboratory studies, it is known that this equipment (earplugs and earmuffs) is effective in reducing exposure to noise, although this effectiveness can lessen under field conditions.^9^

### Hearing protection programs

Studies in the United States indicate that there has been an increase in the use of hearing protection, but that there is still ample room for improvement.^10^ Several factors have been reported to influence the wearing of hearing protection, such as health beliefs, perceived risk, perceived likelihood of risk and comfort of wearing the device.^2,11-13^ Based on these models, several trials have been conducted to study the effectiveness of interventions to influence the wearing of hearing protection and decrease exposure.^14-18^ Until now, there has been no systematic review that summarized the results from these trials.

The aim here was to summarize the evidence for the effectiveness of interventions to enhance the wearing of hearing protection among workers exposed to noise in the workplace.

## MATERIALS AND METHOD

### Selected studies

A literature search covering the period from January 1996 to June 2005 was conducted using the following databases: Medical Literature Analysis and Retrieval System Online (Medline), Excerpta Medica database (Embase), Literatura Latino-Americana e do Caribe em Ciências da Saúde (Lilacs), National Institute for Occupational Safety and Health (NIOSHTIC), International Occupational Safety and Health Information Centre (CISDOC), Cumulative Index to Nursing & Allied Health (CINAHL) and Cochrane Ear, Nose and Throat Disorders Group Specialized Register, the Cochrane Central Register of Controlled Trials (CENTRAL, The Cochrane Library, Issue 2 2005). The following exhaustive list of synonyms for hearing protective devices was used: ear protective device, hearing protective device, hearing protector, hearing protection, ear muff, ear plug, ear defender, protective equipment, (noise, occupational). In addition, the references given in the selected papers were examined, irrespective of the publication year.

Approximately 1500 titles were requested. After the reading of all titles, the reviewers (Regina El Dib and Régis Andriolo) selected 150 potential full articles to be included in the review if they had a randomised design, if they were among noise-exposed (> 80 dB(A)) workers or pupils, if there was some kind of intervention to promote the wearing of hearing protection (compared to another intervention or no intervention), and if the outcome measured was the amount of use of hearing protection or a proxy measure thereof. Of this total, 138 articles were excluded from the review because they were classified as case reports, narrative reviews or letters to editors. Thus, following this assessment of full articles, only 12 publications were considered for inclusion in this review. Six studies were then excluded because they did not meet the inclusion criteria of the review (retrospective studies or non-randomized studies): Davis and Sieber (2002),^19^ Ewigman et al. (1990),^20^ Roeser et al. (1983),^21^ Toivonen et al. (2002),^22^ Walker (1972)^23^ and Williams (2004).^24^ A further three are awaiting assessment because of the poor quality of the reports (Lusk et al. 1999,^14^ Sadler and Montgomery 1982^17^ and Zohar et al.1980^18^). Thus, only two studies (three publications) that met the minimum methodological requirements were included in this review (Knobloch and Broste 1998,^25^ Lusk et al. 2003^15^ and Lusk et al. 2004^16^).

### Methodological quality assessment

In order to assess the methodological quality of the selected studies, a quality assessment list was developed (Appendix 1). The items incorporated are generally accepted methodological criteria. The methodological quality of the trials included in this review was measured using the criteria described in the Cochrane Handbook,^26^ since scales and checklists are not a reliable method for assessing the validity of a primary study.^27^

Two reviewers (Regina El Dib and Régis Andriolo) independently assessed the trial quality of each study, in accordance with this assessment list. Subsequently, disagreements between the examiners, which were small, were discussed to reach a consensus.

### Statistical procedure

Using the available reported data, 2 × 2 tables were constructed to relate determinants to outcomes. In these studies, the outcomes assessed were the proportion of participants who were wearing hearing protection devices relative to the proportion in the control group; intention to use the devices; perceived benefits from using hearing protection; barriers to the use of hearing protection (self-reported use of hearing protection); and awareness of risk. Relative risk (RR) and weighted mean difference (WMD) were used to make estimates of the effects from the treatment in the included studies. Furthermore, the 95 per cent confidence intervals (CI) of these RR and WMD were calculated.

## RESULTS

### Quality assessment

Lusk et al.^15^ and Lusk et al.^16^ described their allocation method as "computer-generated". There was otherwise no mention of allocation concealment. These studies were therefore graded B (unclear) with regard to quality of allocation concealment. Knobloch and Broste^25^ made use of a method to avoid contamination of participants: randomization by clusters. With regard to allocation concealment and generation of allocation, Knobloch and Broste^25^ was graded B, because these details were not described in the paper. Randomization by clusters, as used by Knobloch and Broste,^25^ is thought to be the best way to circumvent the problem of "contamination" inherent to interventions of an institutional nature, in which participants randomized to distinct interventional approaches have the chance to exchange their experiences in a common occupational environment.

Detection bias was present in the Knobloch and Broste^25^ study, since the intervention and data collection were carried out by the researchers. Knobloch and Broste^25^ did not allow for the cluster effect, which could have been estimated by providing intracluster correlation coefficients.^28^ We corrected for the cluster effect by calculating a possible design effect. The intracluster correlation coefficient from other school-based interventions can be estimated as 0.006.^23^ The mean cluster size was 22.1. The design effect (1+(m-1)*r) is thus 1+(22.1-1)*0.006 = 1.1266. We divided the number of events and the sample sizes by the design effect and entered them into RevMan.

In the Lusk et al.^15^ and Lusk et al.^16^ studies, a rigorous method of randomization was used, which was generated by computer and sealed at the time of allocation. Nonetheless, these studies present an indication of selection bias because the trialists did not use a cluster randomization process.

Contrary to what could be expected from the long-term study by Knobloch and Broste,^25^ only 6.4% of the total number of participants in the intervention group and 10.05% of the control group dropped out from the study. The low dropout rates give this study a low risk of bias. The Lusk et al.^15^ and Lusk et al.^16^ studies were considered to present a high risk of bias, because they did not meet the attrition criterion. The withdrawal rate within their relatively short study periods was 53.2%.

### Pooling

The clinical and methodological diversity found in the included studies meant that it was not possible to combine studies in a meta-analysis. Therefore, we only performed representations of meta-analyses, as follows:

#### Participants with personal tailored information versus participants with non-tailored (general) information versus control (commercial videotape) group

**Graph 1:**
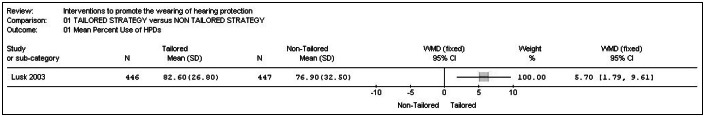
Participants with personal tailored information versus participants with non-tailored (general) information.

**Graph 2:**
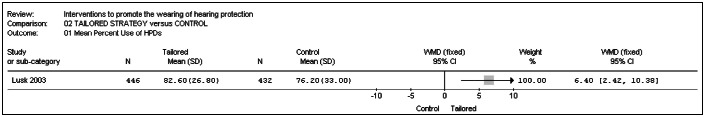
Participants with personal tailored information versus control (commercial videotape) group.

**Graph 3:**
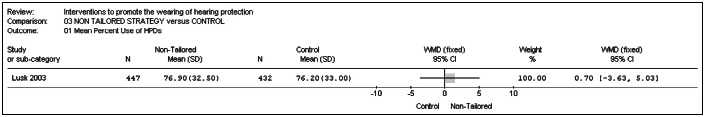
Participants with non-tailored (general) information versus control (commercial videotape) group.

Graph 1 shows a representation of a meta-analysis in relation to the outcome "*Mean percentage of time actually using hearing protection device in required areas, over the preceding week and month*". There was a statistically significant difference favoring the participants who received tailored information rather than non-tailored information in the Lusk et al.^15^ study, with a weighted mean difference (WMD) of 5.70% and 95% confidence interval (CI) of 1.79 to 9.61. Comparing the tailored information strategy versus control (commercial videotape), there was also a statistically significant difference favoring the participants who received the tailored intervention (WMD 6.40%; 95% CI: 2.42 to 10.38), as shown in Graph 2. Finally, there was no statistically significant difference between the non-tailored information group and the control group (WMD 0.70%; 95% CI: -3.63 to 5.03), as shown in Graph 3.

#### Boosters (mailed flyers): All participants with tailored information versus all participants with non-tailored information

**Graph 4:**
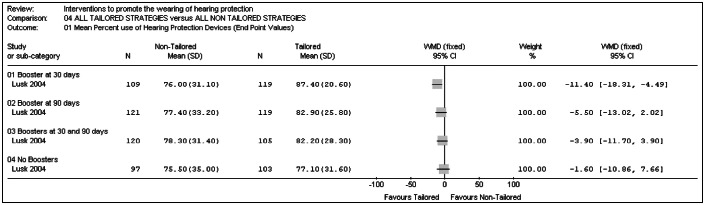
All participants with tailored information versus all participants with non-tailored information.

Graph 4 shows a representation of a meta-analysis in relation to the outcome "*Mean percentage of time actually using hearing protection device in required areas, over the preceding week and month*". There was a statistically significant difference favoring the participants with tailored information in the subgroup that received a booster after 30 days (WMD -11.40%; 95% CI: -18.31 to -4.49; p = 0.001). No difference was demonstrated between the tailored and non-tailored information groups in any of the other subgroups in Lusk et al.^16^ (booster after 90 days; booster after 30 and 90 days; no booster).

#### Boosters: All participants with tailored information versus all control participants

**Graph 5:**
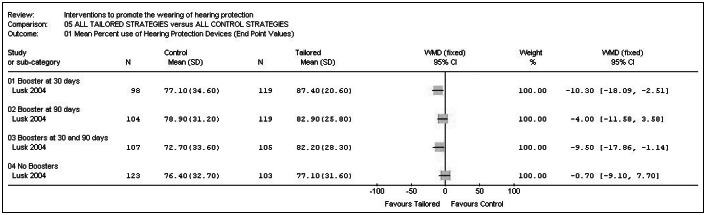
All participants with tailored information versus all control participants.

In relation to the outcome "*Mean percentage of time actually using hearing protection device in required areas, over the preceding week and month*", two particular interventions seemed to favor the participants included in the tailored group over those included in the control group: booster after 30 days (WMD -10.30%; 95% CI: -18.09 to -2.51; p = 0.01) and boosters after 30 and 90 days (WMD -9.50%; 95% CI: -17.86 to -1.14; p = 0.03), as shown in Graph 5.

#### Multicomponent educational intervention versus control (baseline hearing tests and follow-up tests)

**Graph 6:**
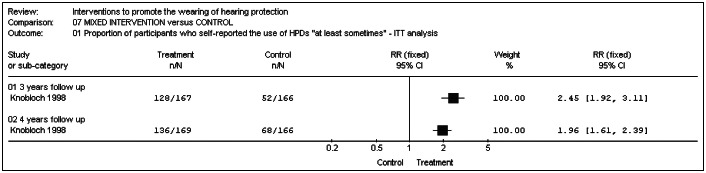
Multicomponent educational intervention versus control (intention-to-treat analysis).

**Graph 7:**
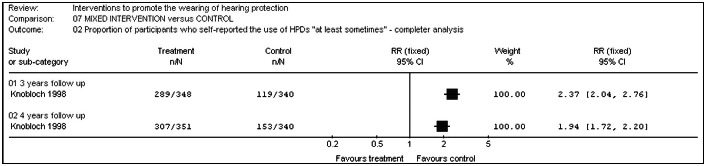
Multicomponent educational intervention versus control (completer analysis).

Knobloch and Broste^25^ measured the percentage of students who used hearing protection devices "at least sometimes". After three years of the study, it was possible to detect a statistical difference favoring the participants in the intervention group over those in the control group (relative risk (RR) 0.42; 95% CI: 0.36 to 0.49; p < 0.00001) (intention-to-treat analysis), as shown in Graph 6. The difference was maintained after four years (RR 0.51; 95% CI: 0.45 to 0.58; p < 0.00001) (completer analysis), as shown in Graph 7. Results from a more rigorous analysis using the intention-to-treat approach revealed that the hearing conservation program trialed by these researchers was effective for both time points, i.e. after three and four years (p < 0.0001). At the start of the study, only 23% of the intervention group and 24% of the control group wore hearing protection "at least sometimes". After three years, this had increased to 83% in the intervention group and 35% in the control group: an absolute difference of 48%.

## DISCUSSION

This systematic review offers up-to-date but limited evidence supported by randomized controlled trials, regarding the effectiveness of interventions to promote the wearing of hearing protection devices. Two of the studies^15,16^ included used the same participants but studied different interventions that both yielded a negative result among workers who had already reported substantial use of hearing protection. The methodological quality of the studies was reasonable, even though there was a substantial risk of attrition bias due to the loss from follow-up of half of the workers. This could imply that only those who were interested and motivated turned up for the post-intervention measurement. Moreover, it could mean that in a highly motivated group of workers it is difficult to increase the percentage of use of hearing protection. However, the risk of hearing loss increases exponentially with the amount of time for which protection is not worn. This means that there is a need to develop interventions that are capable of motivating workers in such a way that the percentage of use will also cover the remaining 20%. Applying the intervention to the whole group, including those that already show perfect behavior in wearing hearing protection, does not seem to be very efficient. It might be worthwhile to explore further the possibilities of targeting the interventions at those that do not use hearing protection.

We found one study that applied the intervention to schoolchildren who were exposed to noise through their work at their parents’ farms.^25^ The methodological quality was reasonable and the number of clusters and the number of participants were sufficient to demonstrate a significant difference. The outcome measurement used in that study (the percentage of participants who used hearing protection devices "at least sometimes") is difficult to interpret in the light of the axiom that hearing protection should be worn for 100% of the time a person is exposed to hazardous noise levels. However, it provides evidence that a school-based program maintained for several years can substantially increase the use of hearing protection, in comparison with a minimal intervention control group. In the United States, it has been suggested that there is substantial room for incorporating occupational health and safety information in vocational training schools.^29^ Furthermore, in occupational health and school settings, cluster randomization seems to be a reasonable approach in designing randomized controlled trials. However, methods to allow for intra­cluster correlation must be used in the analysis of such studies.

There are many qualitative studies that have examined the reasons why workers do not use hearing protection. Comfort^2,30-31^ and the level of enforcement of the requirement to wear hearing protection^11,13,30^ have been reported as factors in not using hearing protection. Several models have been used to explain the variation in the use of hearing protection, such as the health promotion model and the protection-motivation theory.^11,12^ Researchers should make use of these studies to develop new interventions that might be more effective.

There are several studies showing that instructions on how to use earplugs are needed in order to properly insert them. In non-randomized studies that compared the noise attenuation of earplugs used with and without receiving instructions, a significant and important reduction in noise reduction between the groups was found.^22,24^

There is limited evidence that long-term school-based programs can effectively increase the use of hearing protection among students at vocational schools. To date, the limited evidence available does not demonstrate that the use of personalized information to motivate workers to use hearing protection is better than more general information. The limited evidence also does not show that the use of reminders after the intervention increases the use of hearing protection. It could be that this is due to a ceiling effect and only applies in situations in which the majority of workers already use hearing protection.

There are only a few good quality studies evaluating the effectiveness of interventions to promote the wearing of hearing protection devices. More randomized controlled trials are needed. To avoid the risk of contamination, cluster randomized trials are to be preferred. Proper adjustments should be made for the cluster effect and intracluster correlation coefficients should be reported. Future trials should have standardized outcome measurements such the endpoint proportion of participants who wear hearing protective devices in relation to the endpoint proportion in the control group, intention to use the devices, perceived benefits and barriers to the use of hearing protection (self-reported use of hearing protection). Dropouts and losses from follow up should be reported.

## Appendix 1 Data extraction form and methodological assessment list.

**Table:**

EXTRACTION SHEET		
ID – author, year of publication:		
**ACTION** What to ask the author:		
**METHODS**		
1. Design:	
2. Multicenter or single-center:	
3. Period:	
4. Sample size:	
5. Generation of allocation:	
6. Allocation concealment:	
7. Blinded assessment of treatment allocation:	
8. Withdrawals:	
9. Intention-to-treat analysis:	
10. Follow-up:	
**PARTICIPANTS**		
1. N:	
2. Sex:	
3. Age (mean):	
4. Setting:	
5. Inclusion criteria:	
6. Exclusion criteria:		
**INTERVENTION**		
1. Experimental group:	
1.1 Dose:
1.2 Administration:
1.3 Times per day:
1.4 Duration:
2. Control group:	
2.1 Dose:
2.2. Administration:
2.3 Times per day:
2.4 Duration:
**OUTCOMES**		
1. Primary outcome:	
2. Secondary outcome:	
3. Continuous or Dichotomous:	
**NOTES**		
1. Potential for conflict of interest:	
2. Comments:	
**Types of study (randomized or quasi-randomized clinical trial)**		
1. Selection bias – Was allocation concealment adequate?	
A. MET: adequate concealment of allocation;
B. UNCLEAR: not described, not reported;
C. NOT MET: inadequate;
D. Not used.
2. Detection bias – Was there a blinded assessment of outcomes?	
MET: assessor unaware of the assigned treatment when collecting outcome measurements;
UNCLEAR, not reported: blinding of assessor not reported and cannot be verified by contacting investigators;
NOT MET: assessor aware of the assigned treatment when collecting outcome measurements.
3. Attrition bias – Were any withdrawals described?	
MET: less than 20% and equally for both comparison groups;
UNCLEAR: not reported in paper or by authors;
NOT MET: greater than 20% or/an not equal for both comparison groups.
